# Is there a difference in the outcomes of anterior cervical discectomy and fusion among female patients with different menopausal statuses?

**DOI:** 10.1186/s13018-021-02673-2

**Published:** 2021-08-20

**Authors:** Xing-jin Wang, Hao Liu, Jun-bo He, Quan Gong, Ying Hong, Xin Rong, Chen Ding, Bei-yu Wang, Yi Yang, Yang Meng

**Affiliations:** 1 Department of Orthopaedic Surgery, West China Hospital, Sichuan University, No. 37 Guo Xue Xiang, Chengdu, 610041 Sichuan China; 2 Department of Operation Room, West China Hospital, Sichuan University, No. 37 Guo Xue Xiang, Chengdu, 610041 Sichuan China

**Keywords:** Anterior cervical discectomy and fusion, Menopausal status, Fusion rates, Height loss

## Abstract

**Background:**

The surgical outcomes of anterior cervical discectomy and fusion (ACDF) in female patients according to menopausal status remain unclear. The objective of this study was to investigate the differences in these outcomes among female patients with different menopausal statuses.

**Methods:**

Ninety-one patients undergoing single-level or consecutive two-level ACDF with a minimum 12-month postoperative follow-up were included in this study. There were 38 patients in the premenopausal group, 28 patients in the early postmenopausal group, and 25 patients in the late postmenopausal group. The clinical outcomes were evaluated by means of the neck disability index (NDI) scores, Japanese Orthopedic Association (JOA) scores, and visual analog scale (VAS) scores. Radiological parameters included cervical lordosis (CL), the functional spinal unit (FSU) angle, range of motion (ROM) of the total cervical spine, ROM of the FSU, anterior and posterior FSU height, implant subsidence, adjacent segment degeneration (ASD), and Hounsfield unit (HU) values.

**Results:**

All groups showed significant improvements in their JOA, VAS, and NDI scores (*P* < 0.05). The differences in preoperative and final follow-up CL, ROM of C2-7, FSU angle, and ROM of FSU were not statistically significant among the three groups (*P* > 0.05). The anterior FSU height loss rate showed a significant difference (*P* = 0.043), while there was no difference in the posterior FSU height loss rate (*P* = 0.072). The fusion rates in the early and late postmenopausal groups were consistently lower than those in the premenopausal group during the follow-up period. All patients had satisfactory outcomes at the final follow-up.

**Conclusion:**

There were no significant differences in clinical or other related outcomes of single-level or consecutive two-level ACDF in the long term among female patients with different menopausal statuses. However, the early bony fusion rates and anterior FSU height loss rates were poorer in late postmenopausal patients than in premenopausal or early postmenopausal patients. Hence, importance should be attached to the protection of late postmenopausal patients in the early postoperative period to guarantee solid bony fusion.

## Introduction

Cervical degenerative disc disease (CDDD) is a chronic, progressive, and age-related disorder that often presents with mechanical neck pain, radiculopathy, myelopathy, or a combination of these symptoms [1]. With population aging, the prevalence of neck pain has markedly increased over the last 25 years, and CDDD adversely affects quality of life due to its heavy disease burden [1–3]. Anterior cervical discectomy and fusion (ACDF) is one of the most widely used surgical techniques and has been considered the gold standard in the treatment of CDDD [4, 5]. Strong and biomechanical secure fusion after ACDF is critical to obtaining a satisfactory outcome in the long term, and faster fusion is combined with a lower risk of implant failure [6–8]. However, bony fusion is a complex process that is associated with many influencing factors, such as age, sex, bone mineral density, and individual osteogenic capacity [9–11].

In fact, women are more likely to suffer from symptomatic CDDD [12]. According to previous studies, the behavior of periosteal and endosteal surfaces in vertebrae differs between men and women during the aging process, and the bone loss rate is higher in women [13, 14]. Moreover, menopause, namely, the time when menstruation and ovulation cease permanently, is a unique physiological process and an expected life event in women [15–18]. Because of this physiological change, the prevalence of osteoporosis among women aged > 50 years is significantly greater than that in men [19]. However, not all female patients above 50 years old and who require spine surgery have osteoporosis [20]. More importantly, in the early postmenopausal years (< 10 years after menopause), the bone loss rate is nearly triple that of women in their premenopausal years and thereafter decreases to the premenopausal rate for the hip and to zero for the lumbar spine [13]; this indicates that menopause affects bone metabolism but does not necessarily cause osteoporosis and may have an influence on the fusion rates, subsidence rates, and other outcomes of female patients who have undergone ACDF.

To our knowledge, there have been few studies evaluating the surgical outcomes of ACDF in female patients according to menopausal status. The only research on the impact of menopause on single-level ACDF was focused on cages and anterior plates [21]. However, the stability of the Zero-Profile implant system may be inferior to that of the traditional plate-cage construct [22]. Therefore, the bone mass requirements may be different. Considering the paucity of clinical data in this field, a study to verify the surgical outcomes of ACDF in female patients is also warranted. Herein, a retrospective analysis of female patients who underwent ACDF with the Zero-Profile implant system was performed. The aim of the study was to verify the surgical outcomes of ACDF in female patients with different menopausal statuses.

## Materials and methods

### Patient population

This was a retrospective and comparative clinical study. All patients provided written informed consent before their enrollment. Female patients who had undergone single-level or consecutive two-level ACDF with the Zero-Profile implant system (Synthes GmbH Switzerland) from C3 to C7 were enrolled in the study. The inclusion criteria were as follows: (1) female patients (age > 18 years) with radiculopathy or myelopathy due to single-level or consecutive two-level CDDD confirmed by magnetic resonance imaging, (2) no response to conservative treatment for more than 6 weeks, (3) patients who underwent ACDF with the Zero-Profile implant system from C3 to C7, (4) patients with detailed postoperative anteroposterior and lateral X-rays and clinical data, and (5) patients who were followed up for at least 12 months. The exclusion criteria were as follows: (1) patients with a history of trauma, deformity, or tumor or of use of glucocorticoids; (2) previous cervical surgery; (3) spinal deformity; (4) developmental stenosis; or (5) local or systemic infections. The patients were divided into the premenopausal group, the early postmenopausal group (< 10 years since menopause) and the late postmenopausal group (≥ 10 years since menopause) according to whether they had undergone menopause prior to the operation [13]. The study protocol was approved by the Ethics Committee of West China Hospital of Sichuan University.

### Surgical technique

All surgical procedures were performed by the same senior spinal surgeon in our department with a standard, right-side approach. Patients were first put on general anesthesia and the neck was placed in a neutral position. A transverse incision was made at the index level after confirmation of the target vertebral levels. Caspar vertebral distractors were introduced into adjacent vertebral bodies at the index surgical disc level for segmental distraction. The anterior longitudinal ligament, disc tissue, posterior longitudinal ligament, and osteophytes were then completely removed after thorough exposure. After the endplates were carefully prepared, a properly sized Zero-Profile implant filled with a composite synthetic β-tricalcium phosphate bone graft was implanted into the index levels. Then, four fixation screws in the locking head were tightened in an oblique upward and downward fashion. A drainage tube was routinely placed and the deep fascia, subcutaneous tissue, and skin were sutured layer-by-layer. All patients were requested to wear a cervical collar for the first 3 months after surgery.

### Clinical evaluation

All clinical and radiological data were collected preoperatively and at routine postoperative intervals of 3, 6, and 12 months and at the last follow-up. The clinical outcomes were evaluated by the neck disability index (NDI) scores, Japanese orthopaedic association (JOA) scores, and visual analog scale (VAS) scores. The NDI scores were used to assess neck function, the JOA scores were used to evaluate myelopathy status, and the VAS scores were used to assess pain severity.

### Radiographic evaluation

**Fig. 1:**
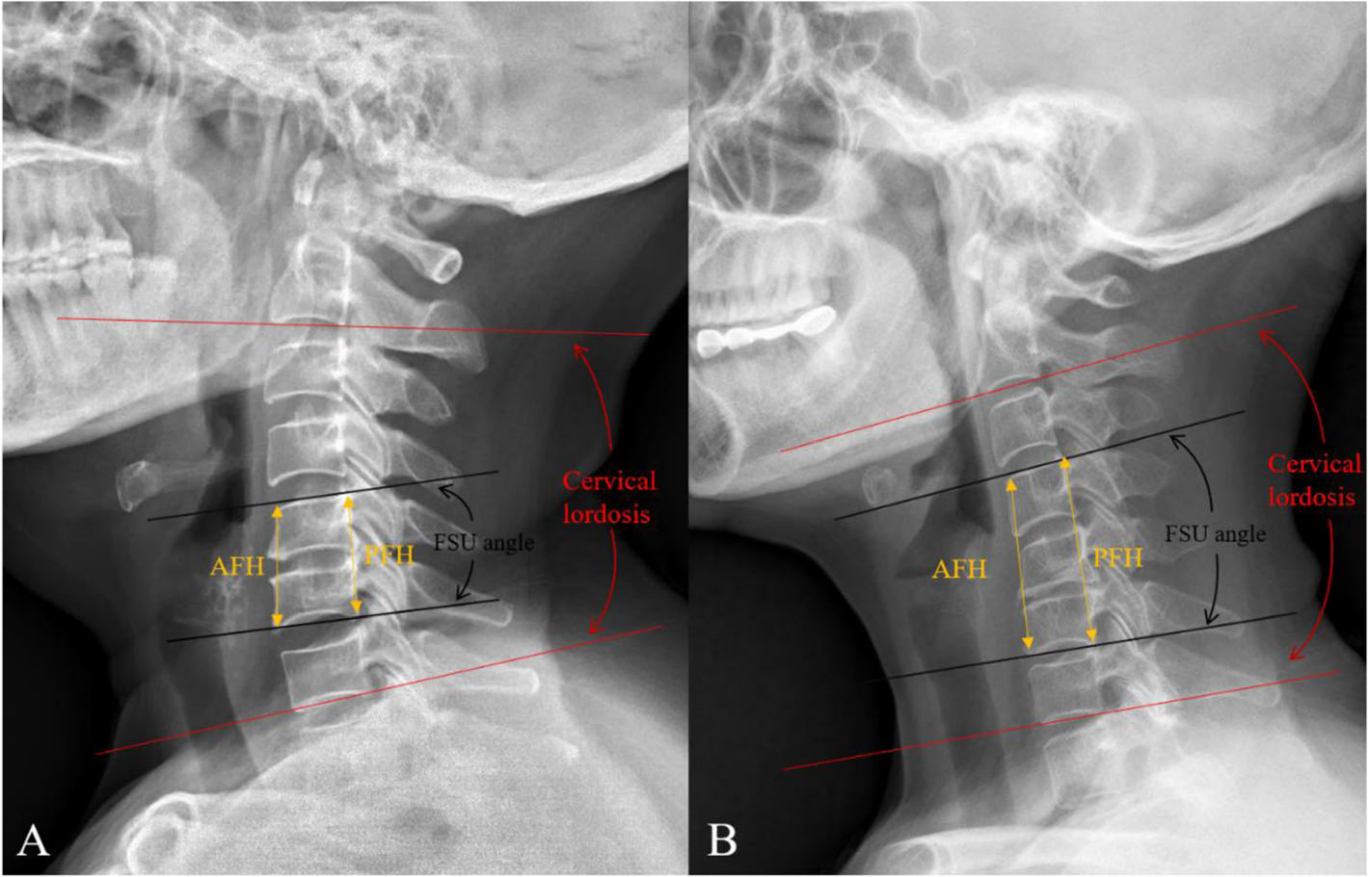
The measurement method for radiological parameters. The angle between the inferior margin of the C2 vertebrae and the inferior margin of the C7 vertebrae was measured as cervical lordosis, while the lines between the superior endplate of the cephalad vertebral body and inferior endplate of the caudal vertebral body were measured as the FSU angle. The FSU height was measured as the distance from the highest portion of the upper end plate of the cephalad vertebra to the lowest portion of the lower end plate of the caudal vertebra in the anterior or posterior FSU. The ROM was the difference in CL and FSU angle between the full flexion and extension radiographs. CL, cervical lordosis; FSU, functional spinal unit; AFH, anterior FSU height; PFH, posterior FSU height

**Fig. 2:**
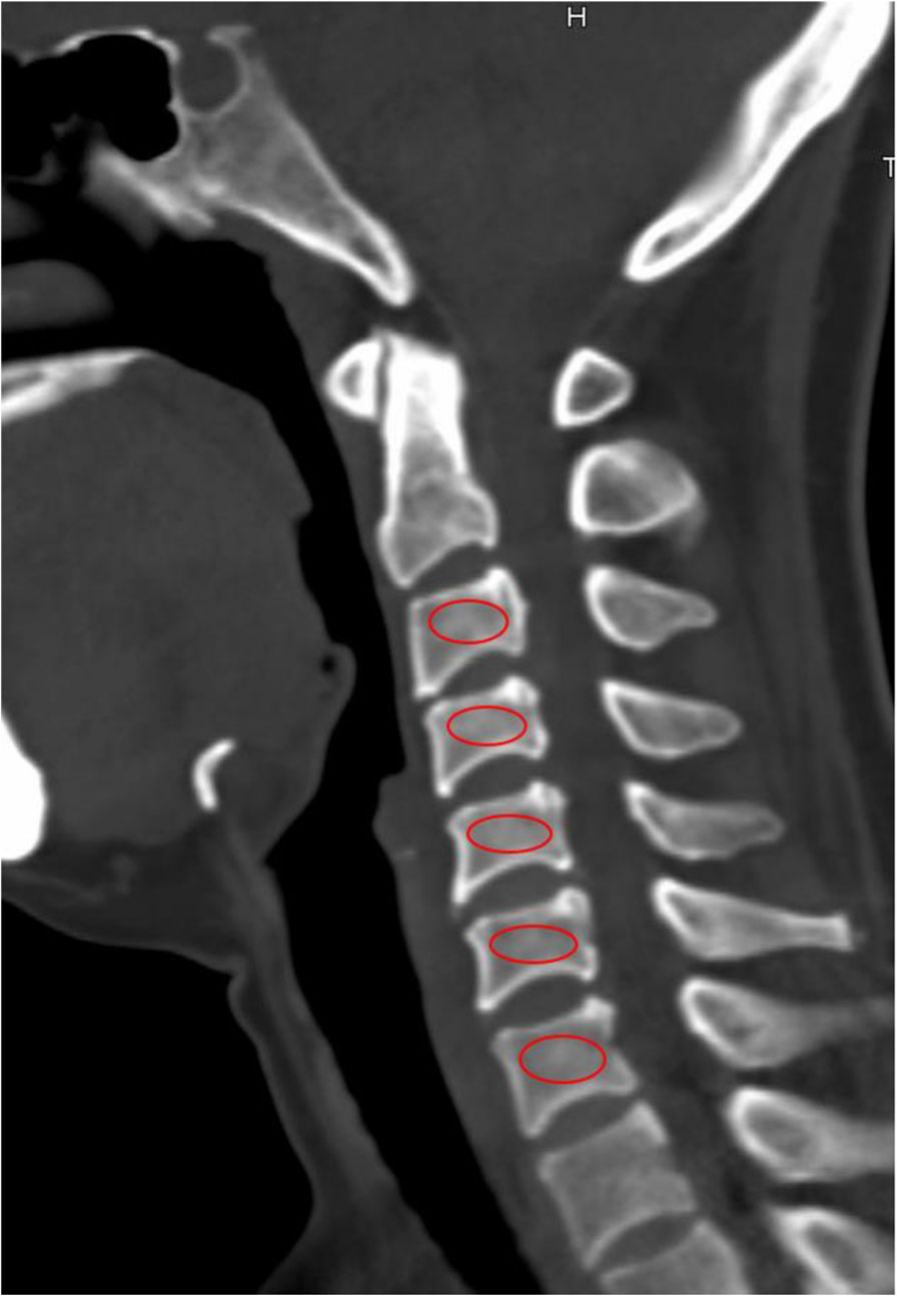
Measurement of HU values in the lateral CT scan reconstruction images

Radiological parameters included cervical lordosis (CL), the functional spinal unit (FSU) angle, range of motion (ROM) of the total cervical spine, ROM of the FSU, anterior and posterior FSU height, implant subsidence, adjacent segment degeneration (ASD), and Hounsfield unit (HU) values (Figs. 1 and 2). CL was defined as the angle between the inferior margin of C2 and the inferior margin of C7 in a neutral position. The FSU angle was formed by the lines drawn at the superior endplate of the cephalic vertebral body and inferior endplate of the caudal vertebral body. The ROM was defined as the difference in CL and FSU angle between the full flexion and extension radiographs. The FSU height was measured on the lateral radiograph as the distance from the highest portion of the upper end plate of the cephalad vertebra to the lowest portion of the lower end plate of the caudal vertebra in the anterior or posterior FSU. The ratios of the height loss of FSU were represented as follows: (height immediately after surgery minus height at last follow-up)/(height immediately after surgery) × 100%. Postoperative implant subsidence was defined by a reduction in ventral or dorsal intervertebral height > 3 mm when postoperative imaging (≤ 1 week) and final follow-up imaging were compared [23]. Bony fusion was assessed by static and dynamic X-ray and computed tomography (CT) imaging of the cervical spine. Successful fusion was determined according to the following criteria [24]: (1) evidence of bridging bone, (2) absence of a radiolucent gap between the graft and endplates, and (3) lack of evidence of > 2° motion. Radiological evidence of ASD was defined as the presence of any of the following findings on the lateral radiograph [25]: (1) new anterior osteophyte formation or enlargement, (2) increased narrowing of an interspace > 30%, and (3) and/or calcification of the anterior longitudinal ligament. The HU measurement for each vertebra (C3–C7) was obtained using an ellipse-type region of interest (≥ 40 mm^2^) excluding the cortical bone margin [26].

### Statistical analysis

All statistical analyses were performed with the statistical program SPSS version 25.0 (SPSS Inc., Chicago, IL, USA). Shapiro-Wilk’s test was used to analyze the data distribution for normality. Variables with a normal distribution are described as the mean (standard deviation, SD), and one-way analysis of variance was performed to analyze whether there were significant differences among the groups. If the variables were not normally distributed, they were presented as the median (interquartile range, IQR), and Kruskal–Wallis tests were used for analysis. For qualitative data, the chi-squared test or Fisher’s exact test was used to compare and presented as the number of patients (%). The paired *t* test or the Wilcoxon signed-rank test was used to compare the preoperative, immediate postoperative, and postoperative data. The results were regarded as significant when the *P*-values were less than 0.05.

## Results

Between March 2011 and March 2020, 91 female patients with a minimum 12-month postoperative follow-up were included in this study. There were 38 patients in the premenopausal group, 28 patients in the early postmenopausal group (< 10 years since menopause) and 25 patients in the late postmenopausal group (≥ 10 years since menopause). The mean duration of follow-up was 19.87 months (range, 12–66 months). Fifty patients underwent single-level ACDF with the Zero-Profile implant, while 41 patients underwent two-level ACDF. The baseline demographic characteristics are shown in Table 1. Age, bone mineral density (BMD), and serum calcium were significantly different among the three groups (*P* < 0.05).

**Table. 1:** Demographic and baseline data

Variables	Premenopausal group	Early postmenopausal group	Late postmenopausal group	*P value*
No. of patients (*n*)	38	28	25	
Age (years)	43.82 (5.32)	54.89 (4.20)	66.76 (4.27)	< 0.001
Intraoperative time (min)	120.00 (120.00–180.00)	120.00 (120.00–120.00)	120.00 (120.00–180.00)	0.399
Blood loss (ml)	50.00 (30.00–50.00)	50.00 (32.50–75.00)	50.00 (30.00–55.00)	0.595
Follow-up period (month)	15.50 (12.00–24.75)	12.00 (12.00–18.00)	12.00 (12.00–24.00)	0.312
BMI (kg/m^2^)	22.40 (20.83–24.56)	23.66 (20.55–25.76)	23.83 (22.27–27.12)	0.165
BMD T value	0.81 (1.16)	− 1.40 (0.98)	− 1.61 (1.09)	< 0.001
Surgery type				0.713
1 level	22	16	12	
2 level	16	12	13	
Serum calcium (mmol/l)	2.26 (2.21–2.32)	2.31 (2.24–2.39)	2.30 (2.25–2.38)	0.027
Serum phosphate (mmol/l)	1.12 (1.05–1.21)	1.18 (1.07–1.33)	1.08 (1.04–1.19)	0.141
Serum magnesium (mmol/l)	0.87 (0.85–0.90)	0.88 (0.83–0.93)	0.89 (0.87–0.92)	0.162

Noncontinuous variables are presented as the number of patients, and continuous variables are presented as the mean (SD) for normally distributed data or as the median (interquartile range) for nonnormally distributed data

### Clinical outcomes

Clinical outcomes, including JOA, VAS, and NDI scores, are summarized in Table 2. At the last follow-up, both groups showed significant improvements in their JOA, VAS, and NDI scores compared with preoperative scores (*P* < 0.05). No significant between-group differences were found regarding the JOA, VAS, and NDI scores, and no implant-related complications were found in any of the groups up to the final follow-up.

**Table. 2:** Clinical outcomes

	Premenopausal group	Early postmenopausal group	Late postmenopausal group	*P* value
JOA scores				
Preoperative	11.00 (10.00–12.00)	11.00 (10.00–12.00)	11.00 (10.00–12.00)	0.163
Last follow-up	16.00 (15.00–16.00)*	16.00 (15.00–16.00)*	15.00 (14.50–16.00)*	0.124
VAS scores				
Preoperative	6.00 (5.00–6.25)	6.00 (5.25–7.00)	6.00 (6.00–7.00)	0.268
Last follow-up	2.00 (1.00–2.00)*	2.00 (1.00–2.00)*	2.00 (1.50–2.00)*	0.405
NDI scores				
Preoperative	30.00 (29.00–32.00)	30.50 (29.00–32.00)	31.00 (29.50–33.00)	0.234
Last follow-up	9.00 (8.00–10.00)*	10.00 (9.00–10.75)*	10.00 (9.50–12.50)*	0.070

*P < 0.05 compared with preoperative value

### Radiological outcomes

Table 3 demonstrates the outcomes and changes during the follow-up period regarding radiographical parameters. The CL and FSU angle showed an improvement at postoperative immediately (*P* < 0.05), while the parameters decreased at the last follow-up. The difference in preoperative and the final follow-up CL, ROM of C2-7, FSU angle, and ROM of FSU were not statistically significant among the three groups (*P* > 0.05). However, compared with preoperatively, the ROM of C2-C7 and FSU were significantly decreased in each group (*P* < 0.05).

**Table. 3:** Radiographical outcomes

Variables	Premenopausal group	Early postmenopausal group	Late postmenopausal group	*P* value
Cervical lordosis (°)				
Preoperative	10.81 (7.14)	12.21 (11.97)	14.04 (9.11)	0.412
Postoperative immediately	12.93 (8.18)	15.89 (8.92)	15.84 (9.02)	0.283
Last follow-up	11.68 (7.56)	12.75 (7.88)	13.78 (7.48)	0.560
ROM C2-C7 (°)				
Preoperative	47.94 (13.11)	42.91 (16.14)	40.76 (14.79)	0.135
Last follow-up	38.90 (7.20)*	36.28 (12.68)*	34.77 (10.83)*	0.267
FSU angle (°)				
Preoperative	2.17 (− 0.58 to 5.56)	4.43 (1.42–7.17)	1.90 (− 2.01 to 6.84)	0.253
Postoperative immediately	4.81 (2.39–10.20)*	5.74 (3.31–12.18)*	4.63 (1.18–11.95)*	0.647
Last follow-up	3.55 (1.35–5.79)* ^#^	4.31 (1.26–6.67) ^#^	2.63 (1.06–5.12)*^#^	0.703
ROM FSU (°)				
Preoperative	10.22 (5.61–17.13)	7.29 (4.12–15.85)	12.50 (8.77–14.83)	0.382
Last follow-up	1.36 (1.11–1.84)*	1.33 (0.85–1.78)*	1.45 (0.93–2.04)*	0.605
AFHR %	2.05 (0.46–2.97)	2.26 (1.04–3.68)	4.32 (0.79–6.79)	0.043
PFHR %	1.41 (0.46–3.80)	1.94 (0.64–4.94)	2.84 (1.71–4.52)	0.187
Subsidence (%)	5.26 (2/38)	10.71 (3/28)	8.00 (2/25)	0.796
ASD (%)	7.89 (3/38)	16.00 (4/25)	25.00 (7/28)	0.072

*P < 0.05 compared with preoperative value

^#^*P* < 0.05 compared with immediate postoperative value

*CL* cervical lordosis, *FSU* functional spinal unit, *AFHR* anterior FSU height loss rate, *PFHR* posterior FSU height loss rate

The height loss rate of anterior FSU showed statistical difference (*P* < 0.05), while there was no difference in posterior FSU (*P* > 0.05). The incidences of subsidence at final follow-up in the premenopausal group, the early postmenopausal group, and late postmenopausal group were not statistically different (*P* > 0.05). Additionally, there was a tendency to have a higher occurrence of subsidence and ASD in postmenopausal group. The mean HU values of each vertebra in the premenopausal group were higher than that in the early and late postmenopausal group and statistical differences were observed (< 0.001) (Table 4).

**Table. 4:** HU values

Level	Premenopausal group	Early postmenopausal group	Late postmenopausal group	*P* value
C3	401.84 (56.68)	313.57 (64.52)	284.60 (56.00)	< 0.001
C4	428.37 (56.71)	321.36 (72.15)	299.44 (59.23)	< 0.001
C5	412.68 (49.41)	319.71 (89.13)	281.48 (70.36)	< 0.001
C6	379.58 (62.66)	286.04 (63.34)	243.20 (57.60)	< 0.001
C7	326.11 (49.16)	245.11 (49.22)	208.48 (53.94)	< 0.001

### Fusion rates

**Fig. 3:**
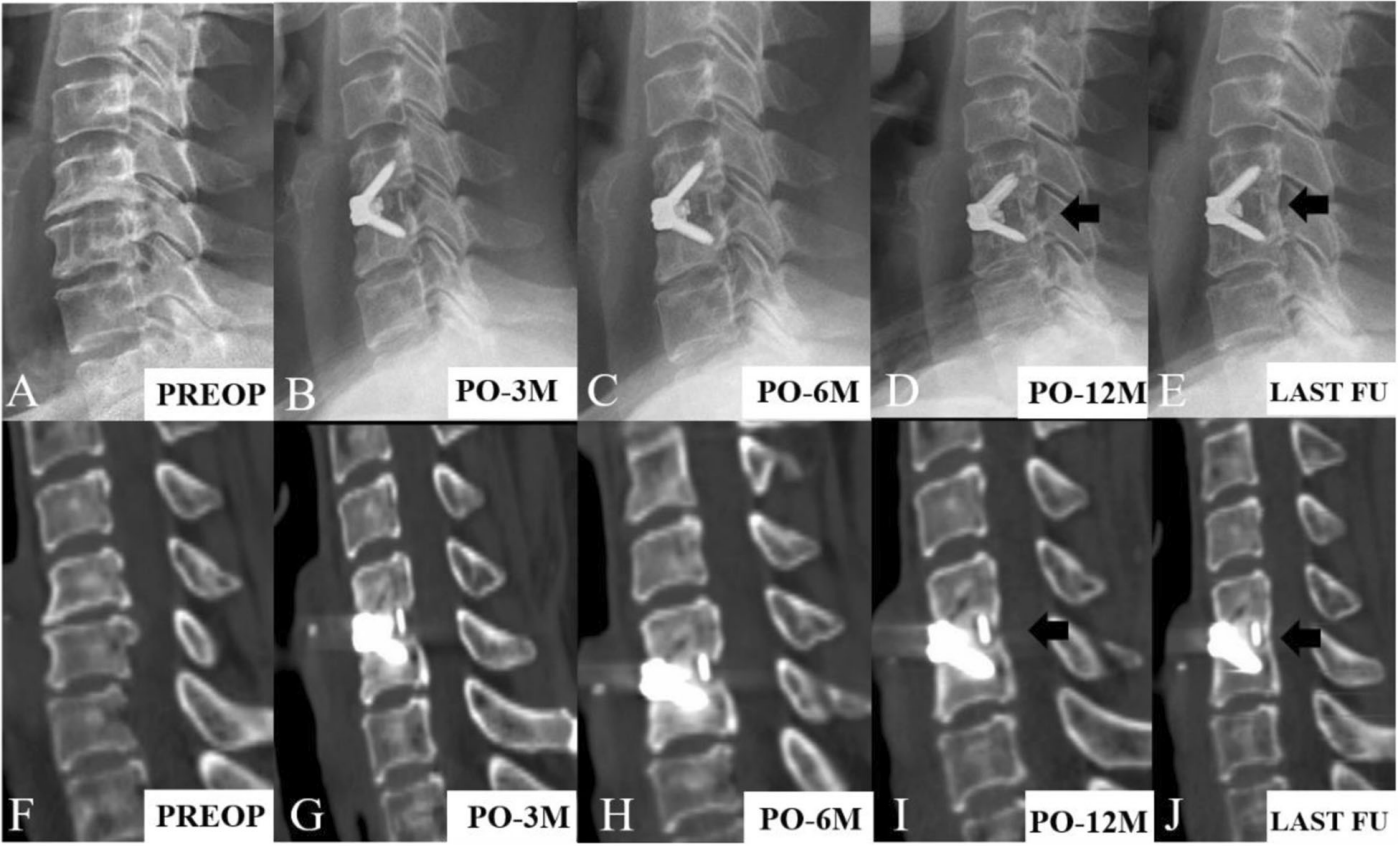
Assessment of fusion status in the lateral radiographs and CT scan images. The bridging bone was observed at the surgical level (black arrow)

The fusion rates of the premenopausal group, early postmenopausal group, and late postmenopausal group during the follow-up period are presented in Table 5. In the premenopausal group, the fusion rates at 3, 6, and 12 months postoperatively and at the last follow-up were 34.21%, 63.16%, 92.11%, and 97.37%, respectively. In the postmenopausal groups, the fusion rates of the early and late groups at each follow-up interval were respectively as follows: 14.29% and 8.00% at 3 months, 35.71% and 32.00% at 6 months, 89.29% and 80.00% at 12 months, and 96.43% and 92.00% at the last follow-up. It is obvious that the fusion rates in the early and late postmenopausal groups were consistently lower than those in the premenopausal group during the follow-up period, and significant differences were observed 3 and 6 months after the operation (*P* < 0.05). In the final follow-up, all patients had satisfactory fusion outcomes (Fig. 3).

**Table. 5:** Fusion rates

	Premenopausal group	Early postmenopausal group	Late postmenopausal group	*P* value
3 months	13/38 (34.21%)	4/28 (14.29%)	2/25 (8.00%)	0.028
6 months	24/38 (63.16%)	10/28 (35.71%)	8/25 (32.00%)	0.023
12 months	35/38 (92.11%)	25/28 (89.29%)	20/25 (80.00%)	0.394
Last follow-up	37/38 (97.37%)	27/28 (96.43%)	23/25 (92.00%)	0.682

## Discussion

ACDF is regarded as the standard surgical treatment for cervical radiculopathy and myelopathy in cervical spine surgery [27, 28]. When used for the appropriate indications, ACDF has a high degree of clinical success and leads to significantly improved outcomes for all primary diagnoses in the long-term follow-up [29]. As the average life expectancy and the proportion of elderly individuals continue to rise, postmenopausal women will become a group with a higher incidence of disease in the future [30]. Thus, the surgical outcomes of ACDF in female patients with different menopausal statuses are worthy of study. In our study, the clinical outcomes of ACDF in all patients showed significant improvements at the last follow-up, and no significant differences were observed among the three groups regarding the JOA, VAS, and NDI scores. The results indicated that female patients with different menopausal statuses can achieve similarly satisfactory clinical outcomes.

**Fig. 4:**
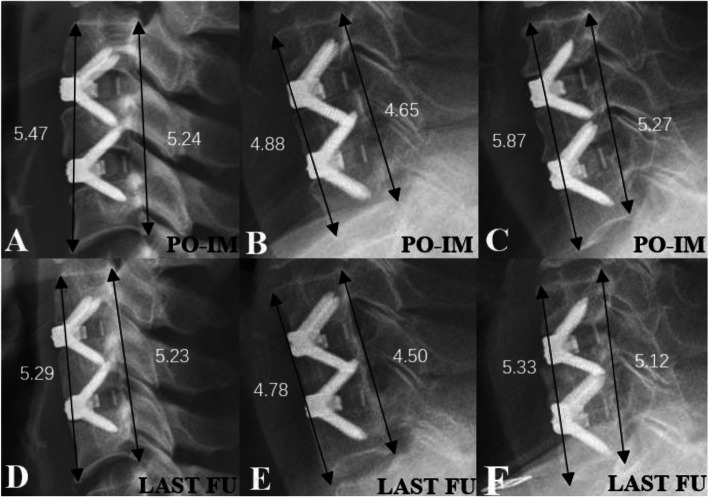
The FSU height (cm) measurement of three patients immediately postoperatively (**A**, **B**, **C**) and at the last follow-up (**D**, **E**, **F**) in the lateral X-rays. The three female patients in the premenopausal group (**A**, **D**), early postmenopausal group (**B**, **E**), and late postmenopausal group (**C**, **F**) underwent consecutive two-level ACDF with zero-profile implants. All groups showed different degrees of height loss of the anterior and posterior FSU and the change in anterior FSU height in the postmenopausal group was significant

The Zero-Profile implant system was used in the surgical procedure. The design philosophy of this implant avoids the need for any additional internal fixation devices and theoretically circumvents the morbidities associated with anterior plating while providing the segmental rigidity necessary for cervical spinal fusion [31]. Regarding the radiological outcomes, the patients who had undergone ACDF surgery with Zero-Profile implants showed an improvement in sagittal alignment immediately postoperative but were gradually lost to follow-up [31–33], which is similar to the results of this study. However, the height loss rate in the anterior FSU was statistically significant (*P* = 0.043), and the rate in the late postmenopausal group was higher than that in the other two groups (Fig. 4). Osteophytes in the patients who need to undergo ACDF are always severe in the vertebral body. To facilitate Zero-Profile implant embedding into the intervertebral space, full decompression is needed during surgery. Thus, the loss of bone mass located at the anterior margin of the vertebral body will have an influence on the support of the upper and lower endplates of the vertebral body for the Zero-Profile implant and contribute to the segmental loss of lordosis [34]. In the normal cervical curvature, the anterior column shares a lower axial load than the posterior column [35]. Nevertheless, CL loss and the FSU angle tend to shift the axial load from the posterior column to the anterior column. The change in cervical alignment potentially increases the mechanical stress in the anterior column and adjacent levels, which will also interfere with the biomechanical environment [36]. Moreover, the BMD and HU values were both inferior in the late postmenopausal group, which may partly lead to changes in cervical alignment and graft subsidence after ACDF [26, 37]. Therefore, the loss of the FSU angle and poor bone mass may explain the loss of anterior FSU height and the degeneration in adjacent levels and potentially have an influence on bone remodeling in the early stage.

Bony fusion is most commonly defined as the presence of trabecular bridging on X-ray or CT imaging and/or the absence of motion on dynamic radiographs at the surgical level after ACDF [38]. In female patients, however, menopause causes an abrupt decline in serum estradiol levels and contributes to a negative metabolic imbalance due to reduced secretion of the ovarian hormones estrogen and progesterone, resulting in bone loss in the skeleton system [15]. The bone loss rate differs between the early postmenopausal years and the late postmenopausal years [13]. Therefore, bony fusion may differ among female patients with different menopausal statuses. Park and colleagues reported that menopause did not significantly affect successful bone fusion in patients who had at least a 12-month follow-up period with single-level ACDF; however, the fusion rates in the early period were not clear, and the implant system included cages and anterior plates [21]. Other studies reported that postmenopausal women may be at higher risk of complications during lumbar spine surgery, but their surgical outcomes were not inferior to those of premenopausal patients [39, 40]. Even though the cervical spine and lumbar spine are two important components in the spinal column and have similar anatomical features, the surgical outcomes for each may differ due to their biomechanics, morphology, and kinematics.

In our study, all groups exhibited satisfactory fusion outcomes at the last follow-up, which corresponded with a previous study [21]; however, the fusion rates in the postmenopausal groups were consistently lower than those in the premenopausal group, and significant differences were observed at 3 and 6 months postoperatively (Table 5). The results suggested that fusion progress was delayed in postmenopausal women, particularly in the late postmenopausal group. In our analysis, late postmenopausal patients had less dense bone, had lower HU values and were older than premenopausal patients, which affected bone metabolism and resulted in negative bone remodeling [26, 39, 41]. Inoue et al. [42] compared the repair process between the metaphysis and diaphysis of ovariectomized and sham mice and found that the fracture healing process was affected from the early phase in the metaphysis and diaphysis in ovariectomized mice. They reported that the inflammation phase was prolonged in the ovariectomized group and that the estrogen sensitivities differed between the sites during the bone repair process, which led to the delay in the osteogenesis process. Duan et al. [14] conducted a cross-sectional study and reported that the diminution in peak vertebral body bone mineral content from young adulthood to old age was higher in women. They also found that the bone loss rate was higher in women due to lower absolute periosteal bone formation in the aging process. However, the exact mechanism needs to be explored in the future.

Given the results, we hypothesized that menopausal status affected bone metabolism and decreased the fusion speed in the early stage postoperatively. Moreover, the height loss rate in anterior FSU was higher in the late postmenopausal group, but it may not have significantly hindered successful fusion in postmenopausal women compared with premenopausal women at the final follow-up.

We speculated the following reasons could be possible interpretations for this phenomenon. Many techniques have been employed to enhance the pullout strength, and rigid fixation instrumentation and techniques can increase the fusion rate [43]. As mentioned above, the components of the Zero-Profile implant system include a titanium alloy plate, a polyether ether ketone polymer cage, and 4 self-locking screws for internal fixation, which will provide the segmental rigidity necessary for ACDF. In our surgical procedure, autologous cancellous bone and β-TCP were used in all patients, which contributed to the formation of bridging bone and promoted fusion in the long term [44]. Additionally, to avoid hyperactivity and injuries after surgery, the patients who underwent ACDF were required to wear a cervical collar for the first 3 months. The collar provided a stable environment for subsequent osteogenesis. Afterward, patients were asked to exercise the neck regularly, and the stress generated during the movement contributed to bone formation. Thus, female patients with different menopausal statuses may have different fusion rates in the early stage, but the fusion rate in the long term is satisfactory.

However, there are some limitations in our study. First, it was a retrospective and unrandomized study from a single institution. Second, only 91 consecutive patients were included, and the patient number and follow-up period were relatively small. Third, data on whether patients received anti-osteoporosis treatment were not collected and analyzed. Thus, a long-term study with a larger number of patients should be performed to further investigate the differential effect of menopausal status on the outcomes of ACDF.

## Conclusion

In the present study, we found that female patients in late menopause were likely to have poorer early fusion rates and FSU height loss rates than premenopausal or early postmenopausal patients, but all patients had satisfactory outcomes at the last follow-up. Hence, we cautiously concluded that there were no significant differences among female patients with different menopausal statuses in terms of the clinical and other related outcomes of single-level or consecutive two-level ACDF. Importance should be attached to the protection of late postmenopausal patients in the early postoperative period to guarantee solid bony fusion.

## Data Availability

Datasets are available from the corresponding author on a reasonable request.

## Abbreviations

ACDF: Anterior cervical discectomy and fusion
CDDD: Cervical degenerative disc disease
NDI: Neck Disability Index
JOA: Japanese Orthopaedic Association
VAS: Visual Analog Scale
CL: Cervical lordosis
FSU: Functional spinal unit
ROM: Range of motion
BMD: Bone mineral density
AFHR: Anterior FSU height loss rate
PFHR: Posterior FSU height loss rate
HU: Hounsfield unit
